# International Committee on Systematics of Prokaryotes: minutes of the open plenary meeting, Tuesday, 17 March 2026, via Zoom

**DOI:** 10.1099/ijsem.0.007156

**Published:** 2026-04-30

**Authors:** Aharon Oren

**Affiliations:** 1 The Institute of Life Sciences, The Hebrew University of Jerusalem, The Edmond J. Safra Campus, 9190401 Jerusalem, Israel

**Keywords:** ICSP, International Committee on Systematics of Prokaryotes, minutes, plenary meeting

## Abstract

An online open plenary meeting of the International Committee on Systematics of Prokaryotes (ICSP) was held on 17 March 2026 via Zoom. To comply with Articles 4(d) and 5(d) (1) of the statutes of the ICSP, the minutes of this meeting are published here.

## Introduction

An open plenary meeting of the International Committee on Systematics of Prokaryotes (ICSP) was held on 17 March 2026 online via Zoom. According to Article 4(d) of the statutes of the ICSP [1], minutes of the plenary meetings of the ICSP are reported by publication in the *International Journal of Systematic and Evolutionary Microbiology* (*IJSEM*). According to Article 5(d), it is the task of the Executive Secretary of the ICSP to submit the minutes of the plenary meetings for publication in the journal. To comply with the statutes, the minutes of this meeting are presented below.

## Welcome and call to order

ICSP Chair E.R.B. Moore called the meeting to order at 09.00 CET. He welcomed all participants to the fifth and last plenary meeting for this term.

## Record of attendance

The meeting was attended by full members and members of the Executive Board: E.R.B. Moore (Chair), C.M. Manaia (Vice Chair), A. Oren (Executive Secretary), P. Nielsen (Treasurer), R. Hahnke (Member at Large), M. Sakamoto (Member at Large), H. Christensen [Vice Chair, Judicial Commission (JC)] and M. Göker (Secretary, JC); co-opted member and member of the Executive Board S.L.W. On (Secretary for Subcommittees on Taxonomy); full members K. Becker, D. Berry, M. Birbir, J.P. Bowman, S. Brisse, Y. Caspar, S.N. Dedysh, B.E. Dutilh, S. Emler, M. Figge, P.-E. Fournier, M. Hamada, M. Janczarek, K. Jangid, M. Joossens, M. Kostovski, S. Korpole, P. Kuhnert, J.S. Lee, W.-J. Li, R. Muñoz, G. O'Hara, U. Reischl, M. Stott, I.C. Sutcliffe, G.Y.A. Tan, A. Táncsics and S. Ventura (also JC member); full members who will join the ICSP on 1 April 2026: D. Cavalieri, L. Cheng, V.R. Kana, W. Ruppitsch, G. Shah, T.N. Ülger and D.-F. Zhang; co-opted members T. Eisenberg, M.-S. Li, M. del C. Montero Calasanz (also JC member), A. Nemec, J. Pieters, A. Sidarenka, J. Tambong and A. Thamchaipenet; JC members C.T. Bull and V. Skye; O. Prakash (Chair, Subcommittee on the Taxonomy of Methanogenic Archaea); K.-i. Suzuki (Chair, Subcommittee on the Taxonomy of the suborder *Micrococcineae*); W.F.A. Teo (alternate for full member L.A. Maldonado Manjerrez); O. Scheler (alternate for full member T. Visnapuu); and guest E.T. Quintana. Apologies were received from D.R. Arahal (member of the Executive Board, Chair, JC); full members S. Butler-Wu, P.S. Cocconcelli, B. Duim, P. Hugenholtz, H. Imachi, T. Lucena, M. Šeruga Musić, Y. Souche, P. Švec and I. Tsitko; and co-opted members R.R. de la Haba, G. Greub (Chair, Subcommittee on the Taxonomy of the *Chlamydiae*) and N. Froude (the Microbiology Society).

## Minute 1. Update of activities of ICSP since the last plenary meeting

E.R.B. Moore explained that this is the fifth plenary meeting organized in the current term, enabling ICSP members, JC members and officers of subcommittees who are not members of the Executive Board to participate in discussions and bring up topics for discussion. The previous meeting was held online on 19 June 2025, and its minutes have been published [2]. He briefly presented a report he had prepared to summarize the activities of the ICSP during the current term that was circulated among all participants before the meeting (see Supplementary material, available in the online Supplementary Material).

## Minute 2. Update of activities of the Judicial Commission (JC)

The 12 current members of the JC are listed at https://www.the-icsp.org/index.php/judicial-commission. During the current term, the JC published four Judicial Opinions (129, 130, 131 and 132) in response to published Requests for an Opinion and has submitted decisions on two more (133 and 134), which are awaiting endorsement by the ICSP.

## Minute 3. Update from the treasurer

The ICSP is holding two accounts in a UK bank, one of which is used for receiving royalties from the Microbiology Society and for running expenses of the Committee, including an annual honorarium to the List of Prokaryotic names with Standing in Nomenclature (LPSN), maintained at the DSMZ; maintenance of the ICSP website; and reimbursements to *IJSEM* Editors for travel expenses to conferences and workshops; the other account holds the historic Skerman bequest to the ICSP to support the award of the C.B. van Niel International Prize. S. Emler asked where the ICSP is legally residing. This is important in case of tax obligations, if applicable. P. Nielsen replied that, financially, the ICSP is registered as a non-profit organization in the UK and has no tax obligations. To his knowledge, no tax declarations are required. It was further noted that the legal residence of the ICSP as a committee of the International Union of Microbiological Societies (IUMS) remains unclear.

## Minute 4. Update from the publications committee and the *IJSEM*

The Impact Factor of *IJSEM* was relatively stable at 2.0 for the past 3 years. Improvement of the impact factor should be a priority for the ICSP Publications Committee in the next term. Typesetting in the *IJSEM* production process remains a continuing problem that should be addressed in the next term.

## Minute 5. The 2025 revision of the International Code of Nomenclature of Prokaryotes

A. Oren, Editor-in-Chief of the International Code of Nomenclature of Prokaryotes (ICNP), explained that after the 2022 Revision of the ICNP [3] was published in May 2023, many significant emendations were approved by the ICSP, including the new Section 10, which includes the formal regulation of the *Candidatus* status in the Rules of the Code, the inclusion of the ranks of kingdom and domain, extensive changes in the Orthography appendix (Appendix 9) and streamlining many other Rules and Recommendations. In January 2025, a formal proposal for the publication of the 2025 Revision of the ICNP was published. After a 6-month open discussion period, the approved version was submitted for publication in November 2025 and published on 9 March 2026 [1]. As no new emendations of the ICNP have been proposed since, the 2025 Revision of the ICNP is expected to serve the community for many years. A. Oren thanked the members of the Editorial Board, and especially M. Göker, co-opted ICSP member M.-S. Li and J. Kelly of the Microbiology Society for the great efforts made during the preparation and production of the document. E.R.B. Moore thanked the Editorial Board of the ICNP for the hard work.

## Minute 6. The 2025 publication of a list of recommended names

M. Göker summarized the status of the work of the Ad Hoc Subcommittee on Mitigating Changes in Prokaryotic Nomenclature (CoMiCProN), formed to address the impact of name changes of prokaryotic taxa in databases, scientific publications and other sources, particularly agencies responsible for establishing protocols and standards for infectious disease control. The committee is composed of members from human medical or veterinary medical microbiology, taxonomists and nomenclature experts, and the proceedings of its meetings are published on the Zenodo platform. CoMiCProN addresses the issue that many practitioners, particularly in the medical and veterinary medicine field, complain about frequent changes in bacterial names. One of the goals of CoMiCProN is explaining that the ICNP does not imply that the last validly published name among a set of synonyms has to be treated as the correct name; a second goal was to provide a list of recommended names (LoRN) of medical importance. The first such list was published in 2025 [4]. Some of the listed names deviate from classifications used by the National Center of Biotechnology Information taxonomy that always treat the last validly published name as the correct name. The LoRN is integrated in the LPSN.

## Minute 7. Impact of the new ICSP statutes

The new version of the ICSP statutes that took effect on 30 September 2025 [5] established changes in member societies' representation. Member societies of the Bacteriology and Applied Microbiology division of the IUMS may now appoint one, two or three full members to the ICSP according to their membership numbers. A. Oren has invited all eligible societies to appoint delegates. As of 15 March 2026, the ICSP has a total of 84 members from 41 countries (49 from Europe, 19 from Asia, 6 from Pacific, 7 from Americas, 1 from Middle East and 2 from Africa): 67 full members, 15 co-opted members and 2 life members. The ICSP noted, with regret, the passing of ICSP life member Professor Jean P. Euzéby (1949–2025). The new statutes extended the voting rights for co-opted and life members. Co-opted members who wish to continue serving for the 2026–2029 term need to be re-elected or reconfirmed by a vote of the full members, from the beginning of the new term. Co-opted members can serve on the Executive Board in all functions except for chair and vice chair. Members of the Executive Board can now serve for two consecutive terms in the same position and one more term in a different function. JC members cannot serve as ICSP voting members at the same time.

## Minute 8. Preparation for the 2026–2029 term

In April–May, the full members will be requested to approve the co-option of candidates who have expressed interest to be elected or continue as co-opted members. In accordance with the statutes, the voting period for all ballots will be 6 weeks. Once the ballots in co-opted members have been finalized, elections will be held for the Executive Board. The new Executive Board will take office on 1 September.

A. Oren, who in the past 24 years has served in the Executive Board for two terms as Chair, two terms as Executive Secretary/Treasurer, and recently two more terms as Executive Secretary, had decided not to volunteer for another term on the Executive Board in a different function. P. Nielsen had notified that he will retire from the Executive Board after two terms as Treasurer, S.L.W. On will retire after two terms as Secretary for Subcommittees, and R. Hahnke will retire from the Executive Board after two terms as member-at-large. E.R.B. Moore thanked the retiring officers. He called upon all ICSP members interested in standing for a position as an officer on the Executive Board to send a letter of intent and provide information about their background.

A new class of 4 commissioners needs to be elected as the 29^th^ Class to replace the 26^th^ Class of the JC on 1 September. In April, a ballot will be held to replace JC commissioner A. Ventosa, who decided to remain a full member of the ICSP and therefore cannot remain a member of the JC. M.-S. Li has expressed willingness to join the JC replacing A. Ventosa. S. Ventura invited A. Oren to join the JC, but A. Oren noted that he prefers serving the ICSP as a full member representing the Israel Society for Microbiology and that he has earlier been an ex officio member of the JC based on an earlier version of the ICSP statutes.

## Minute 9. ICSP online – the website and other online resources

E.R.B. Moore, who manages the ICSP website (https://www.the-icsp.org), stated that the ICSP website has been criticized for not reaching out effectively, particularly to people not involved in the ICSP. Such criticism was coming from colleagues who did not understand the way the ICSP functions. Criticism was noted to arise in part from misunderstandings of the role and function of the ICSP. An example is the paper by de Lorenzo *et al*. [6], published as a commentary in mBio, which falsely claim that the ICSP controls the naming of new organisms and the renaming and the reclassification of organisms. Therefore, it is important to make the website more modern and more informative and interactive to reduce the misunderstandings by the public. He called for volunteers to take over the management of the ICSP website. S. Emler commented that such communication issues are a much more important issue nowadays. The website should provide information on what the ICSP does and also provide useful resources and tools that will be useful for microbiologists. He suggested that two or three persons working together should focus on this task and use professional tools to generate a modern website, possibly assisted by artificial intelligence (AI). M. Göker replied that the ICSP website already has a content management system and technically allows for several persons to obtain access and edit the website. He pointed out that the FAQ document (https://zenodo.org/records/19022845) already explains what the ICSP is doing and what it is not doing; the problem, therefore, is authors who do not actually inform themselves before publishing statements about the ICSP. We need volunteers who regularly update the website. J. Pieters volunteered to become involved in this. R. Muñoz asked whether the IJSEM does have a good website and, if so, whether there is any chance of collaboration. E.R.B. Moore replied that the IJSEM website is managed by the Microbiology Society, and its website focuses on journal publications. V. Skye commented that the Society for General Microbiology maintained and hosted the ICSP website from the 1990s until 2012. W.F.A. Teo extended an invitation on behalf of Bergey’s International Society for Microbial Systematics (BISMiS) to the ICSP to use the BISMiS Zoom and YouTube platforms via the monthly BISMiS Live site (https://bismis.net/bismislive.php) to introduce, share and present the activities of the ICSP, its subcommittees, ad hoc committees and working groups. I.C. Sutcliffe expressed the opinion that the ICSP website is clear, looks quite attractive and contains all of the relevant information about the roles and function and operation of the ICSP. B.E. Dutilh suggested that if we want people to understand what ICSP does, the information should be presented more easily, possibly in a video, instead of a website with much text. He noted that there are people who try to discredit science and/or ICSP, and their audience may not always bother to check whether they are right, or check only superficially. Having a website that is extremely easy to understand may help. E.R.B. Moore commented that until now, the organization of the website has been focused on the ICSP community and scientific aspects and less on reaching out to the general public. R. Muñoz stated that reading long documents with rules and emendations may be cumbersome and confusing to the scientific community. To interpret the code takes an effort, and we should search for ways to make it more user-friendly. S.L.W. On commented that when you type into Google, the first thing that comes up is Google AI, which often leads to wrong results. One should search for ways to design the website so that Google AI picks up the right information, assisting people that are not necessarily well-versed in taxonomy and nomenclature.

The ICSP owns an account on Zenodo, a platform hosted by CERN (the European Organization for Nuclear Research). M. Göker explained that documents posted there are citable with a DOI number. These include the Frequently Asked Questions page (https://zenodo.org/records/19022845), intended to avoid misunderstandings, and meeting minutes of the ICSP Executive Board. Other documents such as minutes of subcommittees on taxonomy could also be posted.

The ICSP has a LinkedIn account (https://www.linkedin.com/company/international-committee-on-systematics-of-prokaryotes) that was opened after we cancelled our account on X/Twitter. Activity on the LinkedIn account has been limited except for the above-mentioned recent case of a publication that misrepresented what the ICSP does, and announcements of ICSP activities, such as the publication of the new revision of the Code. E.R.B. Moore urged everyone to regularly check the LinkedIn account and to post any ICSP-related activity in which the members are involved.

## Minute 10. Activities of subcommittees, working groups and ad hoc committees

S.L.W. On presented an update on the activities of the Subcommittees on Taxonomy. There are currently 18 such subcommittees that are considered active, including the Subcommittee on the Taxonomy of *Borrelia* that was formed in 2023 and the subcommittee on the Taxonomy of *Neisseria* that was formalized in July 2025. E.R.B. Moore emphasized that the Subcommittees on Taxonomy are an important part of ICSP, providing platforms for Subcommittee members to go into in-depth issues of taxonomy and systematics of taxa. S.L.W. On commented that the Subcommittees are the ‘engine’ of the ICSP. However, we are limited in the number of Subcommittees that cover the spectrum of bacterial diversity. Proposals for the establishment of new subcommittees, as outlined in the ICSP statutes, are always welcome. Most subcommittees have published minutes in the *IJSEM*, and one document issued by a subcommittee was published in a different journal, which is allowed under the current statutes of the ICSP. A. Oren commented that such documents must clearly mention that the subcommittee belongs to the ICSP. More than a year ago, S.L.W. On convened a meeting of all subcommittees, and he hopes to convene another such meeting before his term as Secretary for Subcommittees ends in August.

The establishment of an Ad Hoc Subcommittee for Education and Outreach has been proposed. This has been a Working Group over the last years, and S.L.W. On, R. Hahnke and M. del C. Montero-Calasanz have been involved in some activities. M. del C. Montero-Calasanz has organized contests for Spanish high school students for naming novel bacteria with prize money allocated by the ICSP. A. Oren participated in a similar activity with high school students in Austria in a project on the genus *Aquirufa*, and P. Nielsen organized a citizen science project in Denmark some years ago, activating schoolchildren in finding new lactic acid bacteria; he proposed to try to do this again on a global scale. M. del C. Montero-Calasanz also made a proposal for a ‘geocaching’ project involving microbiology. Such activities could well be coordinated by a yet-to-be-established ad hoc subcommittee. E.R.B. Moore is looking forward to receiving proposals and ideas for such a subcommittee, and he encouraged those active in such projects to post relevant information on the ICSP LinkedIn account. D. Cavalieri mentioned the possibility of applying for EU grants dedicated to education and, in particular, citizen science grants to explain the importance of microbial taxonomy. He noted that there is much ignorance on what taxonomy is in an era where everything is based on nucleic acid and protein sequences, and the importance of type strains is often forgotten. A scientific society with the status of a charity can apply for such a grant together with several member universities and act as a grant coordinator according to the European Commission rules.

Information on the CoMiCProN was provided in Minute 6. Colleagues interested in joining this committee should contact M. Göker.

The Working Group on Type Strain Accessibility was tentatively proposed to be changed to an Ad Hoc Subcommittee. E.R.B. Moore explained that this working group deals with issues related to the Convention on Biological Diversity, the Nagoya protocol and Access and Benefit Sharing and special restrictions imposed by some countries on the access to biological material. An example is South Africa, where strict restrictions exist on the distribution of strains, while (as confirmed by J. Pieters), as yet, no restrictions exist on the distribution of digital data. R. Hahnke commented that, in general, the situation with possible restrictions on access to digital sequence information is unclear. S. Emler added that such issues are more driven by politics than by science. In the past, restrictions have been imposed by some countries on the publication of sequences of influenza viruses, thus hampering the development of the seasonal flu vaccine. E.R.B. Moore invited those interested in participating in the working group to contact him.

## Minute 11. Update on the IUMS 2026 Conference, Lisbon, Portugal

The next IUMS conference will be held in Lisbon from 4 to 6 November 2026 in a partnership with the Portuguese Society of Microbiology (SPM). C.M. Manaia, who is a member of the local organizing committee and the delegate of the SPM in the ICSP, explained that on 5 November, a general workshop on taxonomy of prokaryotes, viruses and eukaryotic micro-organisms will be held. It will be preceded by a 1 h session to be sponsored by the ICSP. E.R.B. Moore has already received several proposals for topics and speakers for the ICSP session, and additional suggestions are welcome. The Executive Board will further discuss the content of that session at its upcoming meeting.

## Minute 12. AOB

No additional business was brought up for discussion.

Before closing the meeting, E.R.B. Moore thanked the Officers of the Executive Board for their time and efforts during the last 3 years.

## Closing matters

D. Cavalieri asked what the position of ICSP is in the ongoing debate between the need for type strains and the definition of a taxonomy based only on sequence information. He proposed establishing a working group to publish an opinion paper on this topic. He also mentioned that genomes that were reconstructed *in silico* might be artefacts. H. Christensen replied that in 2020, the ICSP rejected a proposal to allow the use of sequences as type material. Based on that decision, Section 10, Rules 66–73, was added to the ICNP to formalize the *Candidatus* status, to further accommodate not-yet-cultivated prokaryotes. When an organism with a pro-validly published name can later be cultivated, its name can become validly published. B.E. Dutilh commented that we should watch out not to estrange the field of environmental microbiology, which is highly dependent upon nucleic acid sequencing. He has been part of the International Committee on Taxonomy of Viruses, where sequence-based classification is allowed. This would be more difficult for the prokaryotic world unless really good metagenomic assemblies are available, but the pro-valid status sounds like a good idea. M. Göker confirmed that with the new rules of Section 10 of the ICNP, *Candidatus* names can be proposed based on a genome sequence or even a single gene sequence as a nomenclature type. What happens if the same taxon gets later cultivated is also regulated in detail. This system therefore recognizes both the need for dealing with organisms that are, for now, only available as sequences, the continued need to cultivate and the necessity of getting more bacteria in culture and getting deposited in culture collections. This system also caters for the situations in countries that, for legal reasons, cannot deposit type strains in an accessible manner. E.R.B. Moore commented that, unfortunately, the schism between the ICNP and the SeqCode still exists. R. Muñoz asked how many original *Candidatus* names have later been validly published after cultivation of the organism. M. Göker replied that such information can be retrieved from the LPSN database. In many cases, cultivation was achieved by the same group that earlier published the *Candidatus* name. It also happened that another group isolated and cultivated the *Candidatus* taxon later and also reused the name, which is good in terms of nomenclature stability and is now mandatory, based on Section 10 of the ICNP. J.P. Bowman added that he had recently published examples within the family *Oxalobacteraceae*. A. Oren reminded that from 2020 onwards, the list editors of the *IJSEM* have periodically published *Candidatus* lists, and cases of valid publication of names with earlier *Candidatus* status are also listed there. The total number of such genus- and species-level names is ~60.

The meeting was adjourned at 11.00 CET.

## Supplementary material

10.1099/ijsem.0.007156 Supplementary Material.

## Abbreviations

AI: artificial intelligence
BISMiS: Bergey’s International Society for Microbial Systematics
CERN: European Organization for Nuclear Research
CoMiCProN: Ad Hoc Subcommittee on Mitigating Changes in Prokaryotic Nomenclature
DSMZ: German Collection of Microorganisms and Cell Cultures GmbH
ICNP: International Code of Nomenclature of Prokaryotes
ICSP: International Committee on Systematics of Prokaryotes
*IJSEM*: *International Journal of Systematic and Evolutionary Microbiology*
IUMS: International Union of Microbiological Societies
JC: Judicial Commission
LoRN: List of Recommended Names
LPSN: List of Prokaryotic names with Standing in Nomenclature
SPM: Portuguese Society of Microbiology
