# Supplementary material for: International Committee on Systematics of Prokaryotes: minutes of the open plenary meeting, Tuesday, 17 March 2026, via Zoom
Source: Int J Syst Evol Microbiol. 2026 Apr 30;76(4):007156. doi: 10.1099/ijsem.0.007156 (PMC13136530; doi:10.1099/ijsem.0.007156)
Supplement: Supplementary Material. [file ijsem-76-07156-s001.pdf]

Supplementary material

EB-ICSP preliminary Report for the ICSP Plenary Meeting of 17 March 2026

Prepared by E.R.B. Moore and circulated among the members of the ICSP, the Judicial Commission and officers of subcommittees on taxonomy before the plenary meeting

1. Review of current members of the ICSP and its Judicial Commission

At the beginning of the 28th Class of the ICSP term, 01 April 2023, ICSP delegates numbered 31 Full-Members, representing microbiological societies that are also members of the BAM Division of the International Union of Microbiological Societies (IUMS), and 3 Life-Members, appointed for "distinguished service to the ICSP." At the end of the 3-year term (as of 15 March), the number of Societies has increased to 43 from 41 countries and the number of Full-Members has risen to 67 with 15 additional members as Co-opted, "for the purpose of assisting with the work of the Committee" and with 2 Life Members (total ICSP membership: 84). For the next term, 57 Full Members and 2 Life Members have been confirmed, to date.

A current list of ICSP members is found at: https://www.the-icsp.org/icsp-members

The ICSP noted, with regret, the passing of an ICSP Life-Member: Professor J.P. Euzéby (1949-2025).

For a listing of the Officers of the Executive Board of the ICSP, see: https://www.the-icsp.org/index.php/executive-board-ics-p

The term of the Executive Board will end on 31 August 2026. A new Executive Board will be voted on after seating the new Full-Members of the ICSP and voting for any members to be nominated and Co-opted, after 1 April.

The 12 current members of the Judicial Commission are listed at: https://www.the-icsp.org/index.php/judicial-commission

The term of the 26th Class of the Judicial Commission will end on 31 August 2026, with the terms of the 27th and 28th Classes continuing; the 29th Class of four Commissioners should be appointed to start the next term in September. Also, with the resignation of A. Ventosa from the 28th Class, a replacement for his position will be needed.

3. Key activities and achievements of the ICSP in the 2023-2026 term

ICNP: An updated edition of the *International Code of Nomenclature of Prokaryotes* (ICNP, The Code) was published 9 March 2026 in the International Journal of Systematic and Evolutionary Microbiology (Oren et al. 2026, IJSEM, 76:006979; doi.org/10.1099/ijsem.0.006979). "Preface: Although a relatively short time has passed since the publication of the International Code of Nomenclature of Prokaryotes (2022 Revision), … publication of a new revision of the Code is timely, in view of the large number of significant changes that have been approved by the International Committee on Systematics of Prokaryotes (ICSP) …". A three-month ballot for emendation took place August - October 2025 and was passed by the ICSP. To comply with Article 4(d) of the Statutes of the ICSP – 2019 revision, that state that the business of the ICSP should be conducted publicly, the voting was preceded by a 6-month period (December 2024 - July 2025), during which anyone could provide comments to the Editor-in-Chief of the ICNP. In the new Revision, the ranks of Kingdom and Domain were included in the Rules of the Code, the nomenclatural type of a Class was emended to the Genus, clarifications about types of publications to be considered effective publications, clarification of Rule 30, regulation of *Candidatus* names, numerous changes in the Orthography of names, JC Opinions 123-132 issued since the last revision were incorporated. Misconceptions about the mandate of the ICSP remain an issue, with a mistaken perception that the ICSP is directing valid publications of new taxonomic names and reclassifications of bacteria. However, in the last three years, only two editorial articles were published complaining about ICSP interference in prokaryotic taxonomy; the Executive Board is

responding to these issues. Additionally, the ICSP has provided an FAQ page on the ICSP website, which addresses misconceptions about the activity of the ICSP.

ICSP Statutes: "The functions and operation of the ICSP are defined in its Statutes …". A new revision of the statutes was proposed to: empower IUMS Members Societies more representation, based upon the size of membership; modify voting rights of Life- and Co-opted-Members; define separation of powers of different branches of the ICSP; formally regulate Ad Hoc Subcommittees; clarification and streamlining revisions, voting, etc.; change the time that Officers may serve on the Executive Board and provides for Co-opted Members to serve in some Executive Board Officer positions. A three-month ballot for emendation took place January - July 2025 and was passed by the ICSP. To comply with Articles 4(d) and 13(b)(4) of the Statutes of the ICSP – 2019 revision, the voting was preceded by public discussion from March to September 2024. The revised Statutes of the ICSP – July 2025 revision was published on 30 September (Arahal et al., 2025. IJSEM, 75:006918; doi.org/10.1099/ijsem.0.006918).

ICSP Treasury: The ICSP is holding two accounts in a UK bank, one of which is used for receiving royalties from the Microbiology Society (MS) and for running expenses of the Committee; the other account holds the historic Skerman bequest to the ICSP to support the award of the C.B. van Niel International Prize. The recipient of the award is recommended by the ICSP and awarded by the University of Queensland. Activities supported by the ICSP include: an annual Honorarium to the List of Prokaryotic names with Standing in Nomenclature (LPSN), maintained at the DSMZ by M. Göker and colleagues; maintenance of the ICSP website, maintained by Dr. E. Moore; payments for activities by the Working Group on Education and Outreach for projects hosted by ICSP Member, MdelC. Montero Calasanz; and reimbursements to IJSEM Editors for travel expenses to conferences and workshops.

Voting Issues: Voting Members of the ICSP have decided or endorsed issues facing the ICSP. The ICSP held ballots for deciding on 31 issues, including: election of Executive Board Officers and JC Commissioners; co-option of 17 candidates to the

ICSP; approval of 3 JC Requests-for-an-Opinion; 6 emendations to Rules, Recommendations and Appendices the ICNP; revision of the ICNP; and emendation and revision of the ICSP Statutes.

Publications Committee and IJSEM: The Impact Factor of IJSEM was relatively stable for several years, increased dramatically in 2020 but dropped significantly in 2023 (2017, 1.932; 2018, 2.166; 2020, 2.747; 2021, 2.689; 2022, 2.8; 2023, 2.0; 2024, 2.0). The IJSEM remains the official journal of the ICSP and the venue for valid publication of names of prokaryotic taxa. Improvement of the impact (as well as the Impact Factor) should be a priority for the ICSP Publications Committee in the next term. Typesetting in the IJSEM production process remains a continuing problem and improvements have been frustratingly slow to be realized. This should be addressed by the ICSP Publications Committee together with the publications office in the next term.

Subcommittees on Taxonomy: The ICSP currently has 16 active Subcommittees on Taxonomy, which are listed at: https://www.the-icsp.org/taxonomic-subcommittees. The Subcommittees are overseen by the Secretary of the Taxonomic Subcommittees, S.L.W. On (On. 2021. IJSEM, 71(10):005046; doi.org/10.1099/ijsem.0.005046). Many subcommittees convened in-person or online meetings and have published minutes of their meetings during 2023-2026.
Establishment of subcommittees on not-yet-represented taxa should be a priority for the next ICSP term, along with publication of minimal standards for characterizing, classifying and identification of taxa represented by existing subcommittees, as well as members of novel taxa.

CoMiCProN: In January 2024, the Ad Hoc Subcommittee on Mitigating Changes in Prokaryotic Nomenclature was formed (Chair: M. Göker; Secretary: S. Butler-Wu) to “address the impact of name changes of prokaryotic taxa in databases, scientific publications and other sources, particularly agencies responsible for establishing protocols and standards for infectious disease control.” In 2025, a first *List of Recommended Names for bacteria of medical importance* (Göker et al., 2025. IJSEM,

75(10):006943; doi: 10.1099/ijsem.0.006943), with the list incorporated into the List of Prokaryotic Names with Standing in Nomenclature (LPSN) for easy reference.

Nagoya and CBD ABS: Dealing with misconceptions resulting from ongoing challenges presented by the Nagoya Protocol regulations concerning access (distribution) or benefit-sharing (use) remain continuing issues for researchers, culture collections, the IJSEM and for realisation of the Code. The ICSP position is to help support scientists affected by the Nagoya protocol on a case-by-case basis. Because the national legislation has had a profound impact on access and use of genetic resources, including Type strains proposed for novel taxa, a Working Group on type strain availability was formed in the last term to assist in these matters. In the next term of the ICSP, a priority should be placed on the transition of the Working Group to an ICSP Ad Hoc Subcommittee, i.e., for more defined structure and effective functioning.

Important Publications:

- Arahal DR, et al. 2024. The best of both worlds: a proposal for further integration of Candidatus names into the International Code of Nomenclature of Prokaryotes. IJSEM, 74(1):006188; doi: 10.1099/ijsem.0.006188.
- Arahal DR, et al. 2025. Statutes of the International Committee on Systematics of Prokaryotes – July 2025 revision. IJSEM. 75:006918; doi:10.1099/ijsem.0.006918.
- Patrick S, et al. 2025. What’s in a name? Fit-for-purpose bacterial nomenclature: meeting report. IJSEM, 75(7):006844; doi: 10.1099/ijsem.0.006844.
- Freese HM, et al. 2023. The gender gap in names of prokaryotes honouring persons. IJSEM, 73(11):006115; doi: 10.1099/ijsem.0.006115.
- Göker M, Oren A. 2023. Valid publication of four additional phylum names. IJSEM, 73(9):006024; doi: 10.1099/ijsem.0.006024.

- Göker M, et al. 2025. List of Recommended Names for Bacteria of Medical Importance: report of the Ad Hoc Committee on Mitigating Changes in Prokaryotic Nomenclature. IJSEM, 75:006943; doi:10.1099/ijsem.0.006943.
- Jiao JY, et al. 2024. Advancements in prokaryotic systematics and the role of Bergey's International Society for Microbial Systematics in addressing challenges in the meta-data-data era. Natl Sci Rev, 11(7):nwae168; doi: 10.1093/nsr/nwae168.
- Oren A. 2023. Emendation of Rules 8, 15, 22, 25a, 30(3), 30(4), 34a, and Appendix 7 of the International Code of Nomenclature of Prokaryotes. IJSEM, 73(9):006070; doi: 10.1099/ijsem.0.006070.
- Oren A. 2024. On validly published names, correct names, and changes in the nomenclature of phyla and genera of prokaryotes; a guide for the perplexed. NPJ Biofilms Microbiomes, 10(1):20; doi: 10.1038/s41522-024-00494-9.
- Oren A. 2024. Emendation of Appendix 9 of the International Code of Nomenclature of Prokaryotes to regulate the use of connecting vowels in compound names after stems ending in the same vowel. IJSEM, 74(10):006535; doi: 10.1099/ijsem.0.006535.
- Oren A. 2025. Emendation of Recommendation 6(7), Rule 64 and appendix 9 Section D of the International Code of Nomenclature of Prokaryotes to regulate the formation of prokaryote names from personal names. IJSEM, 75(1):006626; doi: 10.1099/ijsem.0.006626.
- Oren A. 2025. Addition of Section 10, Rules 66-73 for further integration of *Candidatus* names into the International Code of Nomenclature of Prokaryotes. IJSEM, 75(1):006638; doi: 10.1099/ijsem.0.006638.
- Oren A, et al. 2024. Proposal to add a Note to Rule 8 of the International Code of Nomenclature of Prokaryotes to clarify the meaning of the term 'stem'. IJSEM, 74(9):006527; doi: 10.1099/ijsem.0.006527.
- Oren A, et al. 2025. Preparing the 2025 revision of the International Code of Nomenclature of Prokaryotes. IJSEM, 75(1):006666; doi: 10.1099/ijsem.0.006666.

- Oren A. 2025. Emendation to Rule 8 of the International Code of Nomenclature of Prokaryotes to clarify the meaning of the term 'stem'. IJSEM, 75(7):006833; doi: 10.1099/ijsem.0.006833.
- Oren A, et al. 2026. International Code of Nomenclature of Prokaryotes, Prokaryotic Code (2025 Revision). IJSEM, 76(3a); doi: 10.1099/ijsem.0.006979.
- Pallen MJ. 2023. Use of connecting vowels after stems ending in the same vowel: a proposal to emend Appendix 9 of the International Code of Nomenclature of Prokaryotes. IJSEM, 73(12):006191; doi: 10.1099/ijsem.0.006191.
- Pallen MJ. 2024. Formation of prokaryote names from personal names: a request of current practice and a proposal to emend Appendix 9 of the International Code of Nomenclature of Prokaryotes. IJSEM. 74(1):006233; doi: 10.1099/ijsem.0.006233.
- Teo WFA, et al. 2026. Notes on the Description of Novel Prokaryotic Taxa. Curr Microbiol. 83(3):152; doi: 10.1007/s00284-025-04619-x.
- Val-Calvo J, et al. 2025. Leveraging the subgenus category to address monophyletic genus over-splitting: illustration with recently proposed *Mycobacteriales* genera. IJSEM, 75(9):006917; doi: 10.1099/ijsem.0.006917.

Conferences and Presentations:

The ICSP hosted a Session of the IUMS Congress 2024 in Florence, Italy, where the 2000 C.B. van Niel International Prize (established in 1986 by V.B.D. Skerman) was awarded by the University of Queensland, Brisbane, Australia, to T. Woyke of the Joint Genome Institute, Berkeley, California, USA; the van Niel International Prize recipient is awarded by the recommendation of the ICSP. The introduction and award was presented by Professor P. Young, University of Queensland. In the ICSP Session, two additional lectures were presented by A. Oren & S. Ventura and by M. Göker.

September 2023, an MS-sponsored Workshop, “What’s in a Name? Fit-for-Purpose Bacterial Nomenclature” was held in Glasgow, Scotland, UK. Lectures by two ICSP members (M. Göker and I.C. Sutcliffe) were presented.

November 2023, the 5th Meeting of BISMiS was held in Guangzhou, China. Several ICSP Members (M. Chuvochina, M. Göker, P. Hugenholtz, K. Jangid, W.-J. Li, E.R.B. Moore, A. Oren, S. Venter) were invited and presented lectures or a workshop.

September 2025, Two ICSP Members, I.C. Sutcliffe and K. Jangid were honoured (Ahmed et al., 2025. doi:10.5281/zenodo.17152607) in a special edition of the Bulletin of BISMiS for their leadership as Presidents of BISMiS (2018-2020 and 2020-2023, respectively).

October 2025, the 6th Meeting of BISMiS was held in Qingdao, China. Several ICSP Members (I.C. Sutcliffe, A. Oren) were invited and presented lectures.

Lectures by ICSP, JC and Subcommittee members were presented at the monthly BISMiS Live online Sessions: A. Oren (December 2023); S.N. Dedysh (February 2024); P. Hugenholtz (March 2024); M. Chuvochina (May 2024); E.R.B. Moore, H. Christensen, M. Göker (June 2024); K. Jangid (August 2024); J.P.W. Young (February 2025); R.R. de la Haba (November 2025).

Judicial Commission: During the 28th Class of the ICSP, the JC published four Judicial Opinions (nrs. 129, 130, 131 and 132) in response to published Requests-for-an-Opinion and has submitted decisions on two more (nrs. 133 and 134), which are awaiting endorsement by the ICSP.

Executive Board: The ICSP EB has met monthly, using GoToMeeting or Zoom, to ensure matters of relevance to ICSP, especially regarding the ICNP and IJSEM are promptly addressed. Minutes are available online at https://www.the-icsp.org/index.php/minutes-reports-2023-2026. The EB has tried to find ways that more equitable representation is exhibited in the ICSP and the EB. Anyone who is

interested in participating more fully in the ICSP may consider to stand for an Officer position in the EB for the next term. All positions are open for candidates from the ICSP to file for intention to stand for office (Note: Co-opted Members may stand for Officer positions with exception of Chair and Vice-Chair) and some of the positions, i.e., Executive Secretary, Treasurer, Secretary for Subcommittees on Taxonomy and one of two Members-at-Large, must change Officers; the incumbent Officers for those positions have reached the maximum term limit and cannot serve in those positions again.
